# Pilot Tests with Waste and Biomass in a 150 kW_th_ Autothermal CLC Unit

**DOI:** 10.1021/acs.iecr.5c01757

**Published:** 2025-10-21

**Authors:** Øyvind Langørgen, Inge Saanum, Roger Khalil

**Affiliations:** SINTEF Energy Research, Sem Saelands vei 11, 7034 Trondheim, Norway

## Abstract

Carbon capture and storage from biogenic feedstocks have recently been emphasized as an important contributor to reach climate neutrality by 2050. Chemical looping combustion (CLC) is an attractive technology in this respect, as it can convert biogenic fuels with high electric efficiency and relatively low capture cost. CLC with biomass has already been demonstrated up to 1 MW_th_ scale, but no pilot demonstrations using waste-derived fuels have been done. In this work, pilot tests with solid recovered waste fuel (SRF) are performed in the 150 kW_th_ scale CLC pilot unit at SINTEF Energy Research in Norway. Tests with woody biomass performed in the same unit serve as a reference. The fuel feeding rate is equivalent to about 115 kW_th_ based on the fuel’s lower heating value. The oxygen carrier (OC) material is Norwegian ilmenite. The results show that the CO_2_ capture efficiency is high, 97.5–98% for the SRF cases. This is even higher than the two biomass cases, which had capture efficiencies of 93.5–95%. The high CO_2_ capture efficiencies are obtained even though this pilot unit has a simplified design without a carbon stripper between the fuel and air reactor. The results indicate that for such reactive and volatile fuels as woody biomass and SRF waste, a carbon stripper might be omitted, saving both investment and operational costs. The fuel reactor (FR) gas conversion efficiency is 80–81% for the SRF cases and slightly lower for biomass with values of 76.5–79.5%. In one test with biomass, ilmenite was mixed with a large share of manganese oxide. This resulted in an increase of the FR gas conversion efficiency of nearly 9%-points. This is most likely due to the higher ability of manganese oxide to release oxygen directly into the FR gas phase, which will ensure a better conversion of volatiles. In essence, the tests show that both SRF and woody biomass are suitable fuels for CLC, that stable autothermal operation can be achieved, and that the carbon capture rate can be high for these fuels even without a carbon stripper.

## Graphical abstract

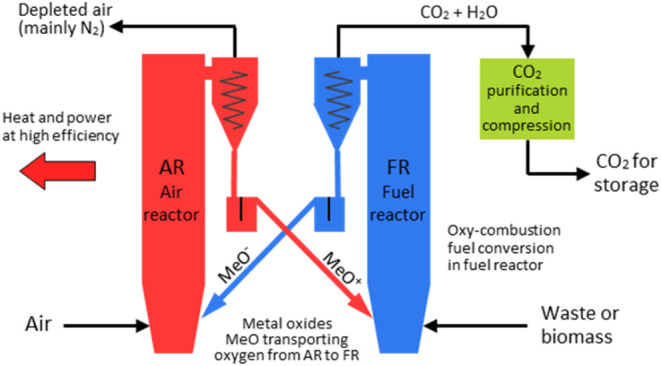

## 1 Introduction

Most emissions mitigation pathways that are likely to limit global warming to 1.5–2 °C by 2100 include CO_2_ capture and storage (CCS) as one of several important measures. A large share of the needed CCS capacity will have to come from carbon dioxide removal technologies, where bioenergy with carbon capture and storage (BECCS) plays a dominant role^1,2^ .

Chemical looping combustion (CLC) is an efficient combustion process for the generation of power and heat from solid fuels, which can deliver a concentrated stream of CO_2_ that can be utilized or permanently stored. It is a two-reactor process where metal oxide particles (the so-called oxygen carrier material) are being oxidized in an air reactor and bring the oxygen to the fuel reactor, where the fuel is converted in an oxy-combustion mode.

Earlier experience with CLC of solid fuels was mainly with coal.^3^ However, the number of CLC studies using biogenic fuels has been increasing due to the interest in BECCS^4–8^ . SINTEF Energy Research has demonstrated autothermal CLC operation with biomass as fuel and ilmenite as oxygen carrier with fuel feed rates above 120 kW_th_.^9^ A general conclusion from these different studies is that CLC is very well suited as a BECCS technology that can achieve high CO_2_ capture efficiency.

Waste-derived fuels with a high share of biogenic content are also relevant for BECCS. Solid waste-derived fuels such as SRF (solid recovered fuel) are already being commercially converted in conventional fluidized bed combustors.^10,11^ Such recovered waste feedstocks have gone through some pretreatment for size reduction, mixing, and homogenization and are well suited for fluidized bed operation.

CLC units are mostly designed as fluidized bed reactors, and pretreated waste-derived fuels might be suitable feedstocks. A challenge of CLC is incomplete oxidation of gases from devolatilization and gasification of the fuel, especially from reactive fuels, such as biomass and waste, containing both biomass and plastics. Another challenge is possible agglomeration and other detrimental effects on the oxygen carrier particles due to constituents in the waste fuel, such as alkali compounds.

In CLC, the air reactor is exothermic, whereas the fuel reactor is generally endothermic but can be slightly exothermic, depending on the type of fuel and oxygen carrier material used. In sum, the energy liberated is equal to the energy in the fuel being fed. The air reactor is also the cleaner reactor of the two. In CLC, it can therefore be a possibility to raise the steam temperatures compared to conventional waste-to-energy plants, without risks for more severe high-temperature corrosion.

In this work, solid waste-derived fuels are being operated in the 150 kW_th_ CLC pilot unit at SINTEF Energy Research for the first time, using ilmenite as the oxygen carrier. Important key performance indicators are evaluated and compared with tests in the same unit using pure woody biomass. The hypothesis is that CLC of solid waste can provide high CO_2_ capture efficiency and fuel conversion, thus being a competitive alternative to BECCS technology.

## 2 Experimental Setup

### 2.1 Pilot Unit Design

The CLC pilot unit at SINTEF Energy Research consists of two circulating fluidized bed reactors, as shown in Figure 1. The air reactor (AR) and fuel reactor (FR) are interconnected through particle loop-seals to ensure there is no gas mixing between the reactors. Only the oxygen carrier (OC) particles are transferred from one to the other reactor. In addition, particles are also transferred from the FR bottom to the AR through the so-called lifter.

**Figure 1:**
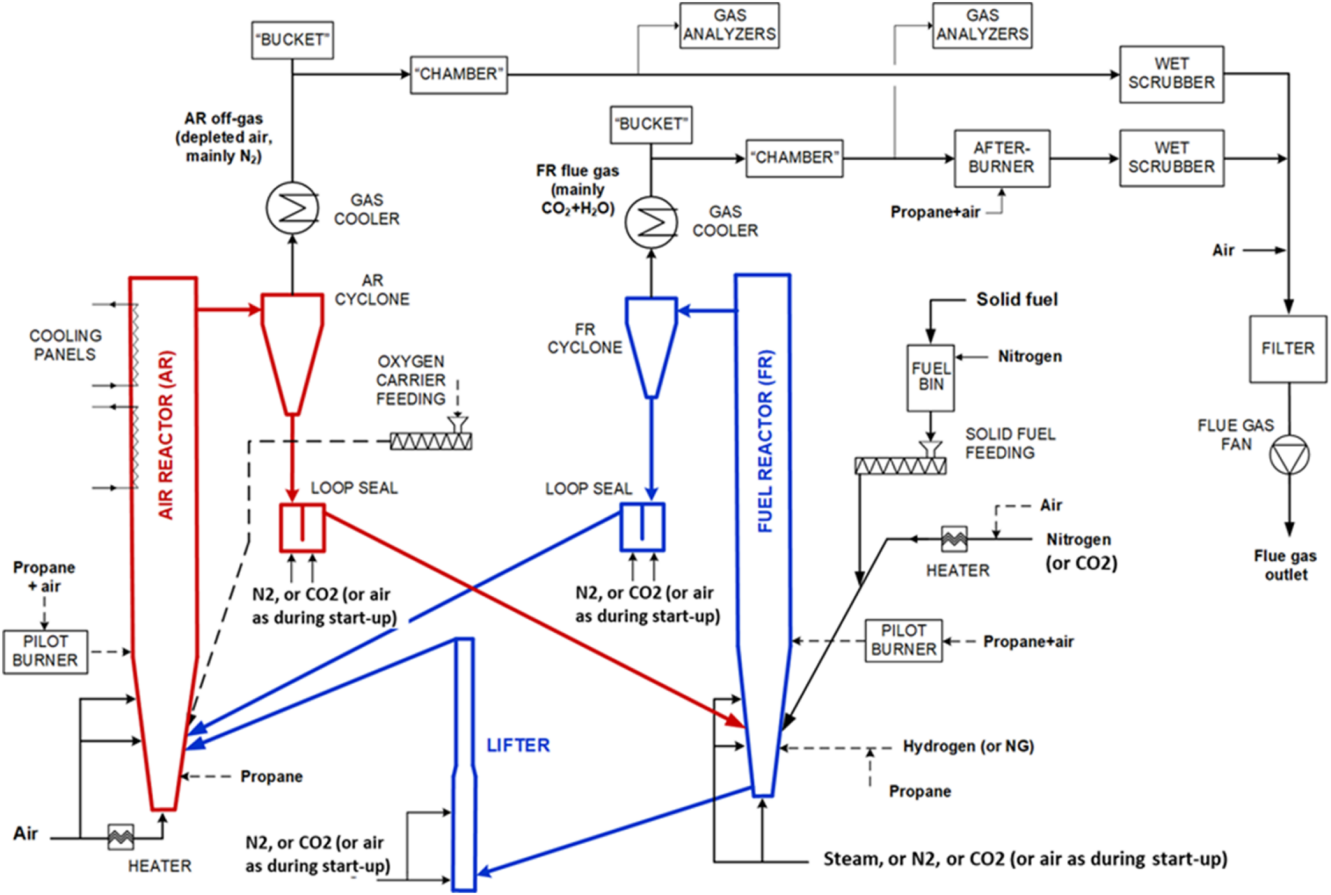
Simplified process flowchart of the CLC pilot unit at SINTEF Energy Research.

The reactors are designed to operate in the fast fluidization hydrodynamic mode with reactor superficial gas velocities in the range 3–5 m/s. Due to the lifter, the FR can also work in a turbulent or bubbling fluidization mode, with almost all of the OC particles being returned to the AR via the lifter. In the tests presented, the lifter was operated in the bubbling regime with a superficial velocity of 0.3–0.5 m/s, and the transport from the FR to the AR was mainly through the lifter.

The system was originally designed for operation on methane as fuel gas, at a maximum fuel power of 150 kW_th_.^12^ Later, it has been modified to allow operation on solid fuels, using mainly biomass as fuel but also some tests with petcoke.^13^ The pilot unit does not contain a carbon stripper between the fuel and air reactor. This simplifies the reactor system and reduces the equipment cost. It also reduces the amount of steam or recycled flue gas that would be needed for fluidization. However, it causes some constraints on what type of fuels can be used. Earlier test results have shown that reactive fuels as woody biomass work well, whereas fuels with lower reactivity, such as petcoke, cannot be operated satisfactorily.^13^

The reactors are arranged in a compact manner, as shown in Figure 2. Both reactors are 6 m tall, of which the first 1 m is a conical bottom section. The remaining 5 m cylindrical sections have internal diameters of 230 mm (AR) and 154 mm (FR). The unit is made in high-temperature steel with external insulation, i.e., no internal refractory is used. Each main part of the unit, i.e., the two reactors, cyclones, downcomers, loop-seals, and the lifter is first covered with a 15 mm layer of Microtherm microporous insulation. Then, a layer of 35–50 mm with Fyrewrap fiber insulation. Due to the compact design, the space and volume between the parts are small, and all the main reactor parts are wrapped within a common fabric from above the conical bottom sections and upward (cf. Figure 2). This is done to minimize the surface-to-volume ratio to reduce heat transfer as much as possible. In an industrial scaled-up CLC unit, the parts will be much larger, having less surface-to-volume ratio and thereby lower heat losses. They will use internal refractory as the main insulation and the parts can be more separated to allow more space for maintenance, etc.

**Figure 2:**
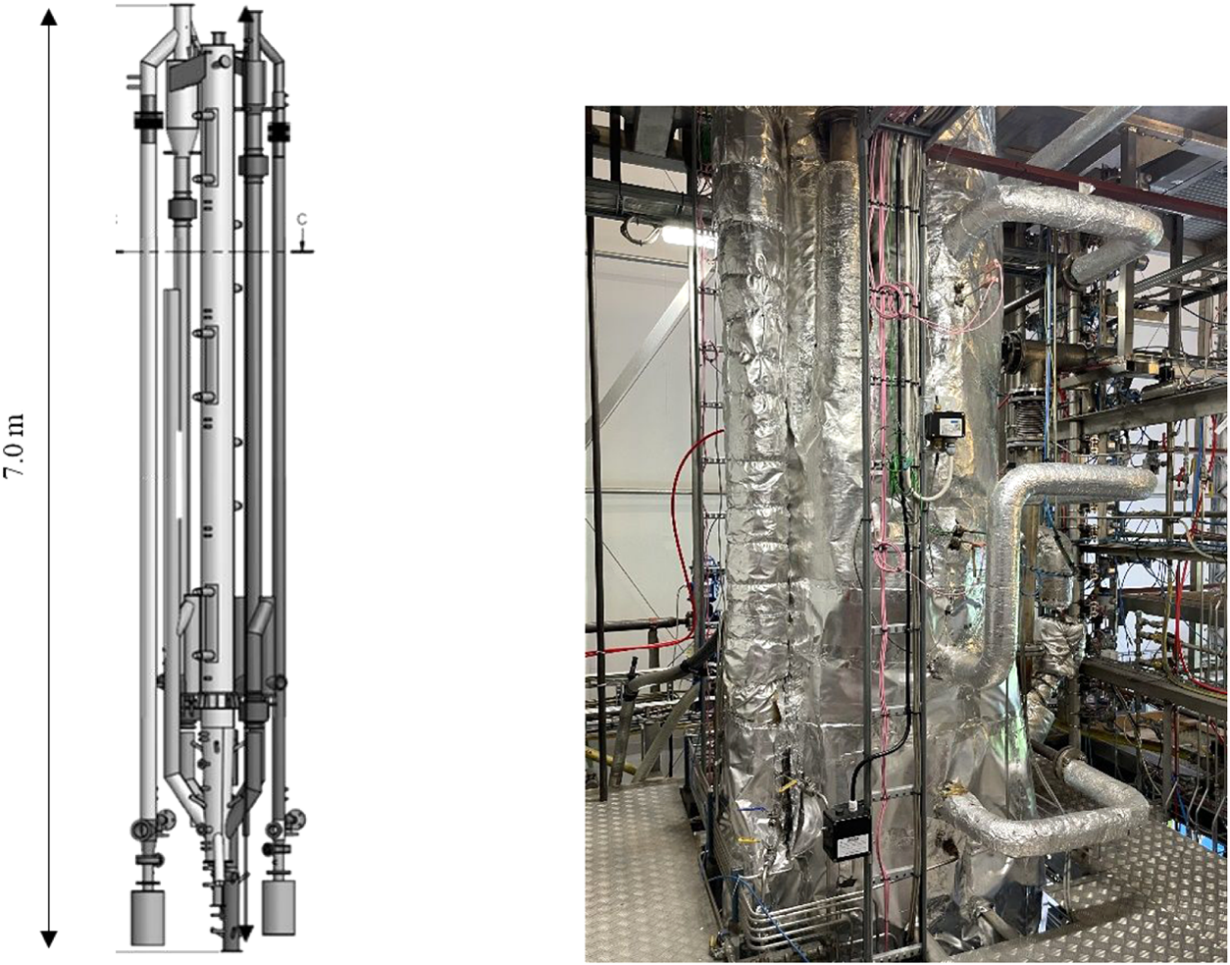
3D model of the main reactor parts and picture of the midsection.

The system has a fuel feeding screw from which the fuel falls into the fuel inlet pipe and is injected into the FR with N_2_ as a carrying gas. There is also an OC feeding screw which makes it possible to refill OC particles during operation.

The unit is equipped with several pressure and temperature transmitters up along the reactors, in the loop-seals, in the lifter, around the cyclones, and in the main inlet streams. The compositions of the outlet exhaust gases are monitored with online gas analyzers. The CO_2_, CO, and O_2_ concentrations of the fuel reactor exhaust are measured with an Emerson Rosemount X-stream IR analyzer. A Varian CP4900 micro-GC instrument is also connected to the fuel reactor exhaust. It measures gases such as H_2_, CH_4_, N_2_, C_2_ hydrocarbons, and helium. Helium is fed to the fuel reactor as a trace gas, and its concentration in the exhaust is used for the mass balance calculation. The micro-GC also measures CO_2_ and CO and therefore serves as a check for the IR analyzer. The air reactor gas outlet is monitored with a Horiba PG-250 IR analyzer, measuring CO_2_, CO, and O_2_ concentrations. During operation, the micro-GC can be switched to measure the exhaust from the AR. This is used to check that no helium leaks from the FR to the AR. If helium is measured in the AR, it means that the loop-seal or lifter is not doing its intended task, which is to just transfer oxygen carrier particles but no gas between the reactors. If some gas can flow together with the oxygen carrier, the bed heights and pressure balance are not correct and the two reactors no longer have fully separate gas atmospheres, as they should. Additionally, the helium feed can be switched to the AR to make further cross-checks of the gas tightness between the reactors.

Each reactor has a “bucket” (see Figure 1) just after the cyclone, where some of the particles leaving the cyclone with the exhaust gas are collected. Downstream this bucket is a larger low-velocity “chamber”, where most of the remaining, and smaller, particles in the exhaust gas are collected. These buckets and chambers have a two-valve system so that they can be closed toward the process and opened at the bottom to be emptied during operation. This is done rather often to estimate OC losses and to calculate the total OC inventory. Particle samples from within the reactors, in the FR afterburner, and in the wet scrubbers are available only when the system is stopped.

### 2.2 The Tested Fuels

A solid recovered waste fuel (SRF) delivered from a Norwegian recycling company was used in the tests. It was originally delivered in loose form, but the fuel feeding system of the pilot unit could not manage such a rather fluffy and low-density fuel. This is due to the relatively small dimensions of the fuel feeding pipe, which also contains some conical reductions. The SRF was therefore pelletized into 6 mm diameter pellets as shown in Figure 3.

**Figure 3:**
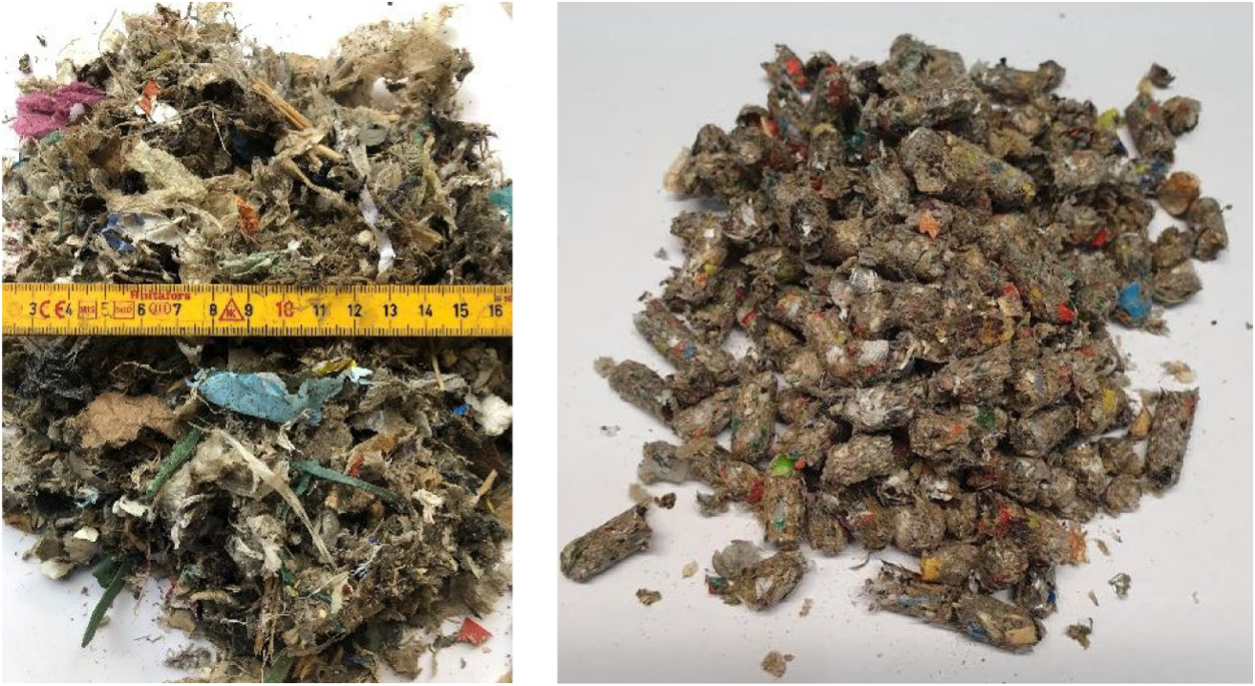
SRF, as delivered in loose form (left) and as pellets (right).

Woody biomass was used as a reference fuel for comparison. Earlier tests have shown that both whole wood pellets and milled wood pellets are well suited for this pilot unit.^9^ The wood pellets are steam exploded, making it brownish, harder, and more water repellent than standard white pellets. The milled variant is just a milled version of the same pellets and includes the fines generated in the milling process. The two woody biomass fuels are shown in Figure 4. The reason to compare the SRF results with both is that the SRF pellets have a much lower density than the wood pellets and that they might disintegrate and fall apart much quicker. A comparison with both whole and milled wood pellets was therefore judged as relevant, as the SRF may disintegrate into smaller particles rapidly in the reactor. Elemental composition and other characteristics for both the woody biomass and the SRF fuels are provided in Table 1.

**Figure 4:**
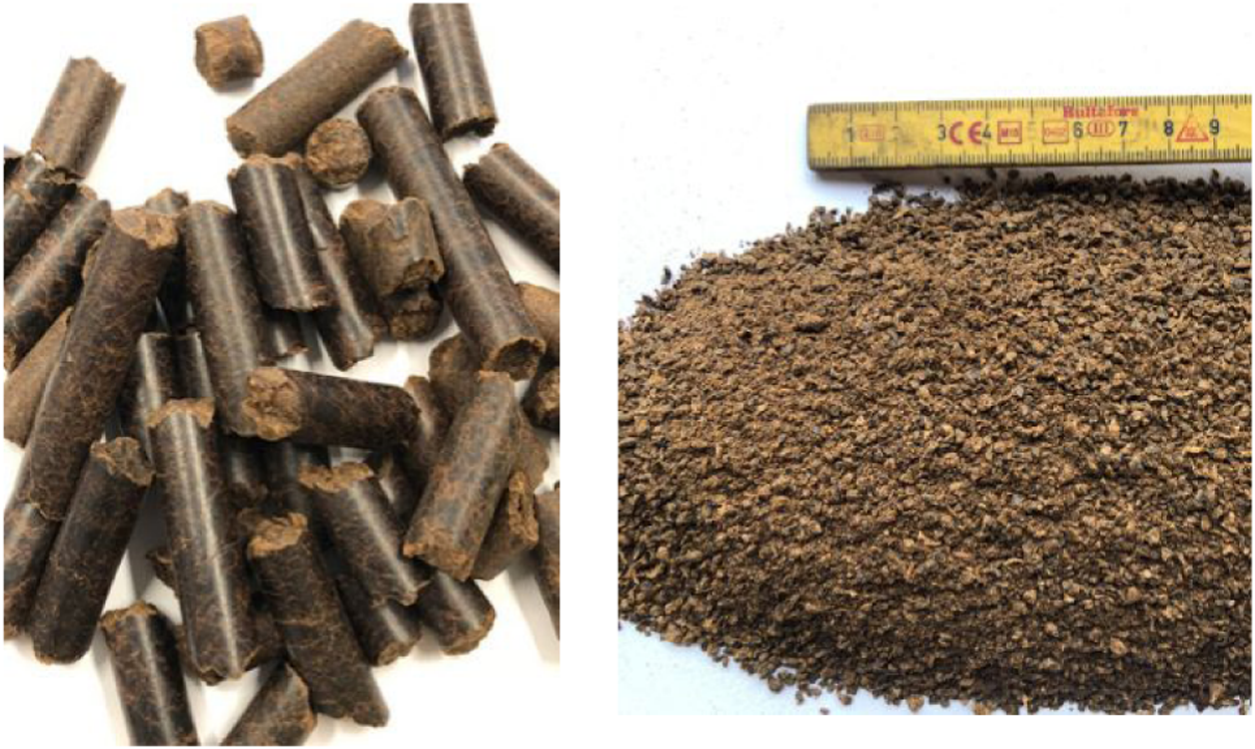
Steam exploded Arbacore wood pellets (left) and milled pellets (right), from ref 9.^9^

**Table 1:** Fuel Analysis Data^a^,^b^,^c^

fuel	unit ^(*)^	woody biomass	SRF
C	wt % a.r.	49.8	50.3
H	wt % a.r.	5.4	6.3
O	wt % a.r.	35.9	22.0
N	wt % a.r.	0.1	1.3
S	wt % a.r.	<0.01	0.39
Cl	wt % a.r.	<0.01	0.83
moisture	wt % a.r.	8.5	6.30
ash (550 °C)	wt % a.r.	0.31	12.6
fixed C ^(**)^	wt % a.r.	18.0	7.5
volatiles	wt % a.r.	73.2	73.6
LHV (MJ/kg)	MJ/kg	18.69	20.52
Φ_0_^(***)^	mol/mol	1.05	1.21
fuel size	mm (pellets)	8 mm × 30 mm	6 mm × 30 mm
	mm (milled)	0.01–3 mm	n/a
bulk density	kg/m^3^ (pellets)	∼650	∼340

a (*) a.r. = as received, i.e., including moisture and ash content.

b (**) By balance with moisture, ash, and volatiles.

c (***) The molar amount of O_2_ needed for full conversion of the fuel per mole of carbon in the fuel.

### 2.3 Oxygen Carriers

The experimental campaign was performed mostly using a Norwegian ilmenite as an oxygen carrier (OC), shown in Figure 5. Ilmenite has been used in many CLC experiments and pilot tests by different research groups.^14^

**Figure 5:**
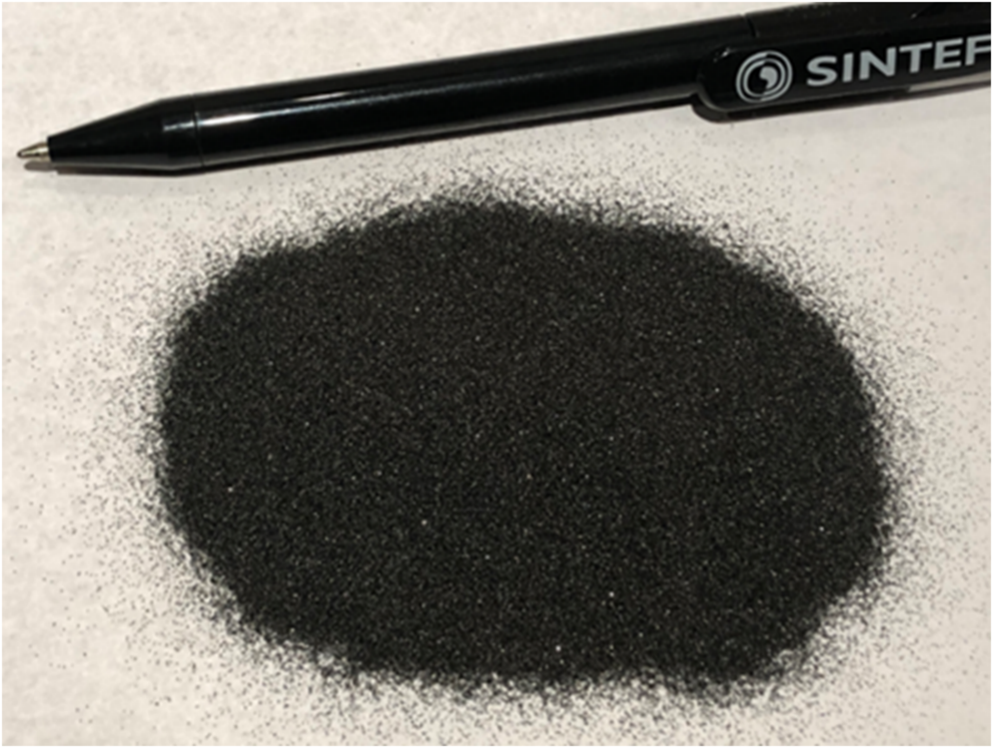
Sample of fresh ilmenite used in the tests.

The ilmenite as delivered has a relatively large particle size distribution (PSD) range of about 10–400 μm. The ilmenite was therefore sieved to a narrower size fraction. Two different fractions of ilmenite have been used in this study, where their PSDs are shown in Figure 6. The smaller and narrower fraction (40–140 μm) was used just in the earliest presented test. It was sieved from the originally delivered ilmenite in two steps, to get rid of both small and large particles. Such small particles result in an increased surface-to-volume ratio compared to larger ones, and possibly higher oxygen transport and less risk of fractures due to mechanical stress from oxidation and reduction. For the later tests, the PSD was widened to 50–250 μm since larger OC particles did not affect the pilot unit operation, and it increased the share of usable OC from the sieving. In fact, the larger size did improve an observed bridging tendency in the AR cyclone. The larger fraction (50–250 μm) was sieved from the same type of ilmenite, but from a batch that had been milled as a preprocess step used in titanium dioxide production. This batch did not contain larger particles, and sieving could be simplified to just removing the smaller particles <50 μm. The tests could have used this milled batch without sieving and instead rely on the CLC reactors to remove fines in the early stage of operation. However, this could have resulted in some operational issues, so it was decided to sieve away the fines before filling up the reactor system. For the much smaller makeup flow of OC during operation, to compensate for particle losses, it is expected that the unsieved batch, as delivered, can be used since it will not significantly increase the share of small particles in the system.

**Figure 6:**
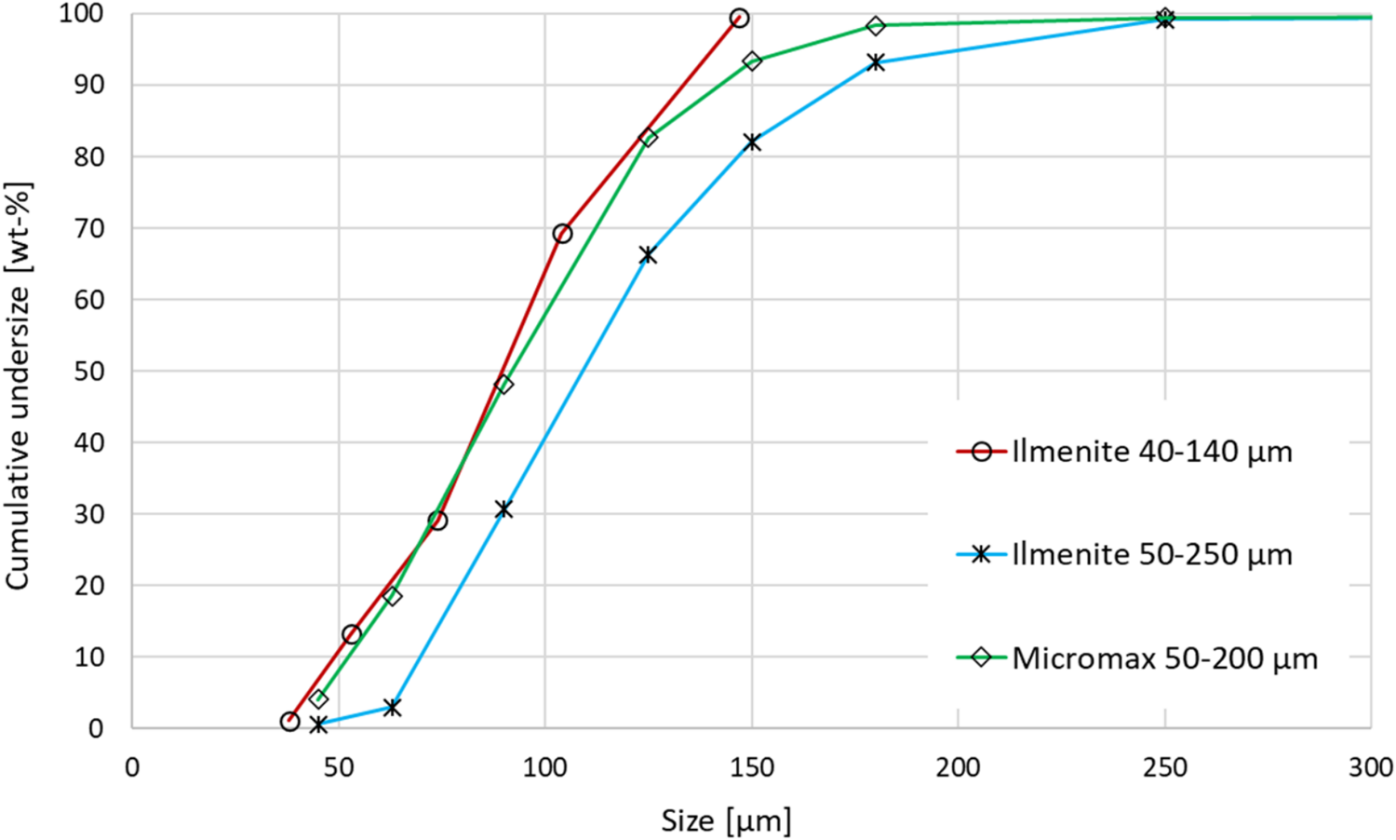
Particle size distribution of the fresh oxygen carriers before experiments.

In addition, one biomass test was performed with ilmenite mixed with manganese oxide material. The reason is that manganese oxide has a CLOU (chemical looping oxygen uncoupling) effect that is higher than that of ilmenite. i.e., it has a higher ability to release oxygen directly to the gas phase in the FR. This can provide an increase in the fuel conversion efficiency, especially when converting fuels with a high share of volatiles, such as biomass and SRF. The manganese oxide used is Micromax. It is a manganese tetraoxide (Mn_3_O_4_) based material. The PSD of Micromax is presented in Figure 6 together with the ilmenite samples.

The Micromax was mixed with the ilmenite fraction 50–250 μm in a mixture consisting of 70 wt % Micromax and 30 wt % ilmenite. They were mixed inside the reactors during fluidization, so the PSD of the mixture before the experiments is not available. As seen from Figure 6, the Micromax has a particle size that is about 20–25 μm smaller than the ilmenite. It has a poured bulk density of about 1915 kg/m^3^, while the ilmenite samples are heavier having a poured bulk density in the range 2500–2600 kg/m^3^ when being fresh and unused. After some time in CLC operation, the density of the ilmenite will decrease significantly, to about 1600–1700 kg/m^3^ bulk density. The particles from the two materials will then be of rather equal size distribution and density, leading to comparable fluidization and entrainment behavior.

### 2.4 Data Evaluation

#### 2.4.1 Fuel Reactor Carbon Conversion

The fuel reactor (FR) carbon conversion *X*_C_ represents the percentage of carbon fed with the fuel that is leaving the FR in gaseous form after devolatilization, gasification, and combustion. The remaining carbon will leave in particulate form, either following the FR outlet gas, or following the OC over to the AR. The FR carbon conversion, which should be as high as possible, is calculated as

1 $$X_{C}=\frac{\left(x_{CO_{2},\mathrm{FR}}+x_{\mathrm{CO},\mathrm{FR}}{+x}_{CH_{4},\mathrm{FR}}+{2x}_{C_{2}H_{y},\mathrm{FR}}\right)F_{\mathrm{out}}^{\mathrm{FR}}}{F_{C,\mathrm{feed}}^{\mathrm{FR}}}$$

where *F*_out_^FR^ is the total dry molar flow rate of gas out of the FR (kmol/h), and *x*_*i*,FR_ is the mole fraction of carbon-containing gaseous species *i* in the dry gas out of the FR. Finally, *F*_C,feed_^FR^ is the flow rate of carbon that is fed to the FR with the solid fuel (kmol/h).

#### 2.4.2 Oxygen Demand and Fuel Reactor Gas Conversion Efficiency

The flue gas from the fuel reactor (FR) is mainly CO_2_ and H_2_O but will also contain some unburnt compounds (CO, CH_4_, H_2_, others). A small stream of additional O_2_ downstream of the FR, in the so-called oxygen-polishing step, will then be needed for full fuel conversion. An important performance parameter is, therefore, the fuel conversion efficiency within the fuel reactor, which should be as high as possible to reduce the need for additional O_2_.

The oxygen demand represents the ratio of the additional oxygen needed to completely oxidize any unconverted gases leaving the FR, to the stoichiometric amount of oxygen needed to fully oxidize all of the combustible gases that are released in the FR through devolatilization or gasification. The oxygen demand should be as low as possible and is calculated as

2 $$\Omega_{OD}=\frac{0.5x_{CO,FR}+{2x}_{CH_{4},FR}+0.5x_{H_{2},FR}+{3x}_{C_{2}H_{y},FR}}{\Phi_{0}\left(x_{CO_{2},FR}+x_{CO,FR}+x_{CH_{4},FR}+{2x}_{C_{2}H_{y},FR}\right)}$$

where Φ_0_ represents the molar amount of O_2_ needed for full conversion of the solid fuel per mole of carbon in the solid fuel. The C_2_H_*y*_ is included in the equation as the measured C_2_H_2_ + C_2_H_4_ (measured together by the GC) was a little too high to ignore, even though it is often ignored in the literature.

The fuel reactor (FR) gas conversion efficiency, η_gas_, is just one minus the oxygen demand and should be as high as possible

3 $$\eta_{gas}=1-\Omega_{OD}$$

The oxygen demand and FR gas conversion efficiency calculated this way is related only to the gas phase. A real fuel conversion efficiency would also take into account the solid carbon particles, leaving the FR as unconverted fuel. If there is any such carbon loss, it will cause a slight increase in the oxygen demand compared to the oxygen demand Ω_OD_ as calculated by eq 2.

#### 2.4.3 CO_2_ Capture Efficiency

In CLC, it is the carbon leaving with the FR exhaust that can be captured. The CO_2_ capture efficiency η*_CC_* is therefore equal to the ratio of carbon leaving out with the FR exhaust to the carbon amount fed with the fuel. Any carbon lost to the AR will immediately be converted to CO_2_ and leave with the AR exhaust, causing a reduction in the capture efficiency. Also, CO_2_ as fluidization gas should be avoided in locations where the gas could leak to the AR. The CO_2_ capture efficiency is calculated according to

4 $$\eta_{\mathrm{CC}}=\frac{F_{C,\mathrm{feed}}^{\mathrm{FR}}-F_{C,\mathrm{out}}^{\mathrm{AR}}}{F_{C,\mathrm{feed}}^{\mathrm{FR}}}$$

where *F*_C,feed_^FR^ is the flow rate of carbon that is fed to the FR with the solid fuel (kmol/h), and *F*_C,out_^AR^ is the carbon leaving through the AR. The definition in eq 4 is based on that carbon leaving the FR in other forms than CO_2_ (gaseous species as CO, CH_4_, etc., and solid carbon particles) is also considered as captured carbon because it will be converted to CO_2_ in a downstream oxygen-polishing step. Any remaining char particles could be considered stable for storage.

#### 2.4.4 Oxygen Carrier Circulation and Inventory

The oxygen carrier (OC) circulation rate is an estimate of the OC mass flow transported to the AR reactor. This value is equal to the OC circulation rate between the reactors. This value is calculated using an analytical riser mass flow expression involving the pressure difference (Δ*p*) between the two upper pressure transducers (at 3 and 6 m height), the difference in height between the transducers (Δ*h*), the superficial gas velocity (*u*_0_), the terminal velocity of the OC particles (*u*_t,_ 0.4–0.8 m/s,), the reactor riser flow area (*A*), and gravitational acceleration (*g*)­

5 $${ṁ}_{\mathrm{riser}}=\frac{A}{g}\frac{\Delta p}{\Delta h}\left(u_{0}-u_{t}\right)$$

The real circulation rate was less than this. Values of 10–30% of the theoretical one have been reported^15,16^ .

The oxygen carrier inventory is estimated by calculating the average solid–gas mixture density between subsequent pressure transducers using the pressure difference (Δ*p*) and height between the transducers (Δ*h*). The densities are multiplied by the volume between the pressure transducers and summed to give the OC inventory of each reactor and the lifter.

The fuel reactor specific OC inventory (kg/MW_th_) is the mass of the OC inventory in the FR, divided by the fuel feed expressed in thermal power based on its lower heating value (LHV). A high value is generally preferable, since it means that more OC is available to convert a certain amount of fuel.

### 2.5 Test Procedure

The pilot unit is operated during the daytime, resulting in a heat-up sequence for each test day. This sequence is shorter during consecutive days of testing since the unit retains some heat overnight. An overview of a whole test day is presented in Figure 7. For this test, the CLC operation was performed with SRF pellets. During the heating sequence, air is injected into both reactors, for fluidization and combustion. First, the air is heated by the 30 kW electric heaters, one for each reactor. The pilot burners in each reactor are also ignited, providing about 5 kW_th_ each. When the temperature in the bottom parts of the reactors reaches about 350 °C, gaseous fuel injection through the fuel lance in each reactor is carefully started, using the pilot burners to ensure that the fuel is ignited. Propane is used in the air reactor, and hydrogen is used in the fuel reactor. Hydrogen is beneficial since its high flame speed makes sure the flame is anchored close to the fuel lance positioned down in the OC bed, thus efficiently heating the oxygen carrier particles. A hydrogen supply line is currently not installed for the air reactor.

**Figure 7:**
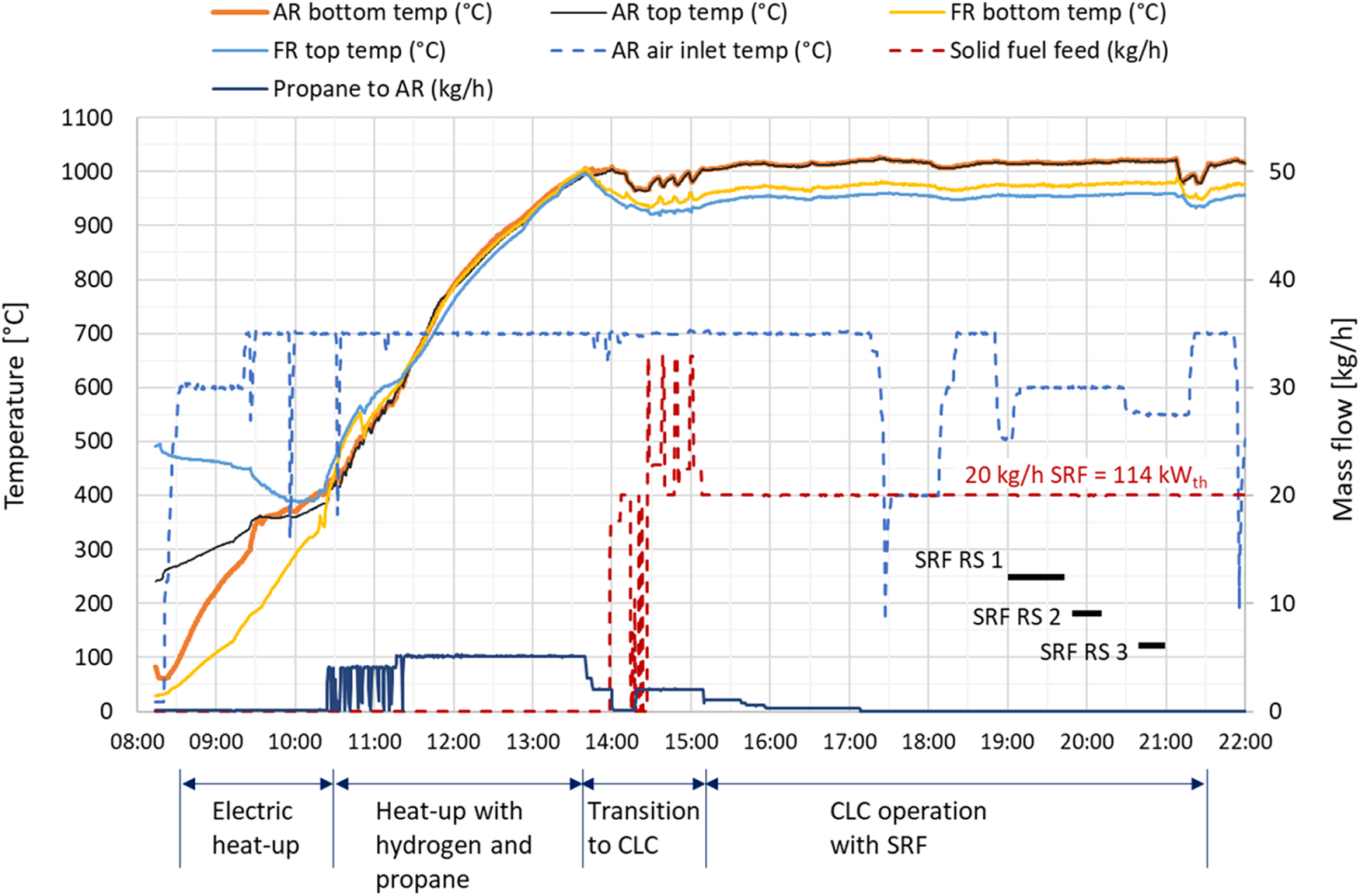
Overview of operational sequence during one whole test day with SRF.

In case the unit is started with fresh ilmenite, the ilmenite will need some preoxidation and activation time. Preoxidation will be fulfilled during the heat-up with air and combustion with high air excess ratio. For ilmenite activation, the fuel is shifted to biomass pellets when the temperature is above 900 °C. Air is still injected in both reactors, but the amount of air to the FR is gradually decreased to substantial levels under the stoichiometric level. This period is maintained for about 2 h to ensure complete activation of the ilmenite OC.

When the heating and activation sequence is finalized and all reactor temperatures have reached about 1000 °C, the air flow to the FR is turned to zero to get into CLC mode. Propane to the AR is shut off; however, it might be necessary to reintroduce propane also during CLC mode, after operational upsets when the process needs to regain the heat loss and get back to the same temperature levels. When the temperatures in the AR and FR reactors remain stable and high, without any external heating, and without any propane injection to the AR, the unit is considered to be in autothermal CLC mode, except for the electrically preheated air entering the AR.

During CLC operation, all conditions (temperatures, reactor pressures, inventories, feed flows, etc.) are kept as constant as possible, except for small changes made from time to time to try different operating conditions and evaluate the performance. Data are evaluated from time-averaging the measured quantities over time intervals of at least 10 min. In addition, the process should be in stable operation without any changes for at least 10 min prior to the averaging period. These periods are rather short; however, since the unit is made of steel with only external insulation, the thermal inertia is low compared to, for example, refractory-lined units. If the temperatures do not change very much, all reactor quantities adjust to new settings in just a few minutes. For periods with a longer time of stable operation, the time used for averaging is increased accordingly.

## 3 Results and Discussion

### 3.1 Test Overview and Operating Conditions

An overview of the test cases, including fuel and OC data, is given in Table 2. The first two cases are provided from ref 9.^9^

**Table 2:** Overview of Test Cases, Oxygen Carrier, Fuel, and Test Case Date and Averaged Period^a^

test case	oxygen carrier, type and size (μm)	fuel, type, and size	date and averaged time period
wood pellets (from ref 9)	ilmenite 50–250	wood pellets ArbaCore	15.11.2022 16:30 – 17:29
milled wood pellets (from^9^)	ilmenite 40–140	pellets ArbaCore, milled and unsieved	06.12.2019 14:07–14:31
SRF pellets 1	ilmenite 50–250	SRF pellets testpoint 1	03.11.2022 19:00–19:39
SRF pellets 2	ilmenite 50–250	SRF pellets testpoint 2	03.11.2022 19:52–20:08
SRF pellets 3	ilmenite 50–250	SRF pellets testpoint 3	03.11.2022 20:42–20:57
wood pellets Mn + Ilm. ^(*)^	micromax 50–200 + Ilmenite 50–250	wood pellets ArbaCore	18.11.2022 13:52–14:11

a (*) Oxygen carrier in this case is a mix of 70 wt % Micromax and 30 wt % ilmenite.

The main operating conditions of the tests as well as the measured temperatures and gas concentrations are shown in Table 3. All values are averaged over the time intervals given in Table 2. The AR riser mass flow is the theoretically calculated value according to the expression given in Section 2.4.4. The nitrogen from the FR originates from the nitrogen used to fluidize the loop-seals and the lifter and the nitrogen used as a carrier gas to help feed the solid fuel. Nitrogen is also used as the main fluidization gas in the FR in all cases except the milled wood pellet case, where steam was used. However, the need for additional fluidization gas in the FR is limited due to the high level of generated volatiles when wood or SRF fuels are used. These fuels also contain high amounts of hydrogen that will form water vapor, thus supporting gasification reactions, without the need to introduce additional steam as a fluidization gas.

**Table 3:** Main Operating Conditions, Measured Temperatures, and Gas Concentrations

	main operating conditions				temperatures				gas concentrations (on dry gas)							
test case	solid fuel power (kW_th_)	air excess (λ)	AR riser mass flow (kg/s)	FR spec. inventory (kg/MW)	FR bottom (°C)	FR top (°C)	AR average (°C)	air preheat (°C)	FR CO_2_ (vol % dry)	FR CO(vol % dry)	FR H_2_ vol % dry)	FR CH_4_ (vol % dry)	FR C_2_Hy (vol % dry)	FR N_2_ (vol % dry)	AR CO_2_ (vol % dry)	AR O_2_ (vol % dry)
wood pellets	105	1.3	2.8	190	959	937	1009	675	37.3	7.3	1.9	3.0	0.5	49.8	0.8	10.5
milled wood pellets	120	1.0	3.5	128	964	954	997	860	43.9	6.5	1.8	3.2	0.4	44.0	1.5	6.5
SRF pellets 1	114	1.2	4.7	222	974	955	1015	587	33.9	5.0	1.2	2.8	0.6	57.3	0.3	9.6
SRF pellets 2	114	1.2	4.3	222	976	956	1017	600	37.4	5.1	1.2	3.0	0.6	53.7	0.3	9.7
SRF pellets 3	114	1.2	4.4	212	978	959	1019	550	37.7	5.1	1.1	2.9	0.5	53.6	0.4	9.7
wood pellets Mn + Ilm	105	1.1	2.1	134	971	954	1013	385	41.6	4.0	0.7	2.3	0.2	50.0	1.1	5.9

The air reactor temperatures are even, with a difference between the top and bottom of just 2–3 °C. Table 3 therefore shows just the average AR temperatures. For the fuel reactor, the variations are larger, with differences in top and bottom temperatures generally about 20 °C. Both FR temperatures are therefore given in Table 3, since this might affect the different fuel reactions, mainly gasification reactions in the bottom part and gas burnout in the upper part.

A full-scale industrial CLC unit must be able to operate fully autothermal. However, many research units are electrically heated to maintain the necessary reaction temperatures. The present pilot unit can operate in autothermal mode except from preheating of air to the AR, but due to its small size in comparison to industrial units, it operates just at the limit of autothermal and it is not as straightforward to set the reactor’s temperatures as it would be if the unit was electrically heated. Both the heat and mass balance must be fulfilled through the operational set-points, just as it will be in a real unit. For this reason, there is some variation between the operating conditions of the different cases.

The share of the particle flow that runs through the lifter is dependent on the fluidization velocity and the pressure difference between the reactors and is not straightforward to calculate. Figure 8 shows the reactor pressure profile of the reactor system for one of the tests. Since the pipe down to the lifter can be considered a downward extension of the FR, the lifter bottom pressure is drawn together with the FR pressure at negative 0.51 m.

**Figure 8:**
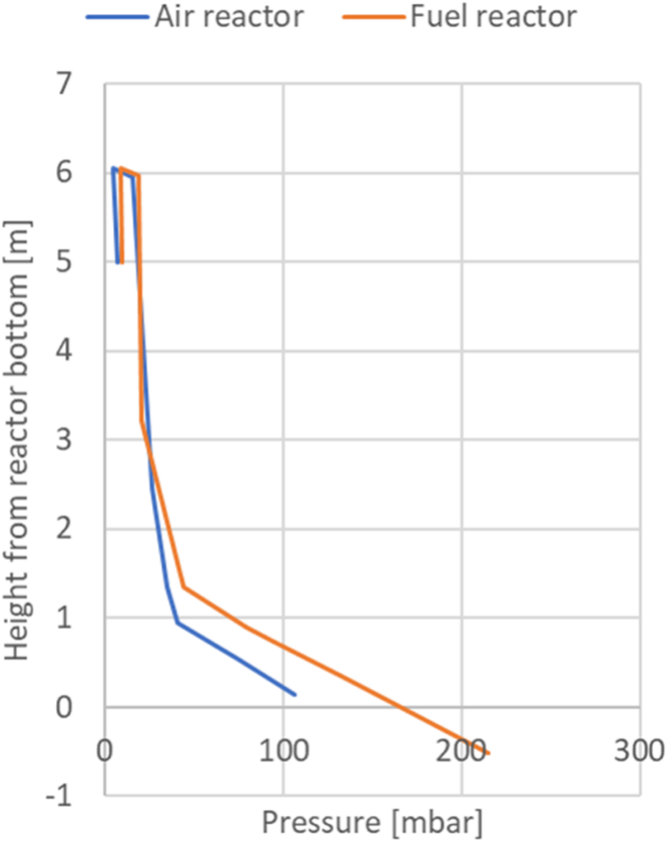
Reactor pressure as a function of height in the AR and FR, test “SRF pellets 1”.

The pressure gradient in the upper part of the reactors is higher in the AR compared to the FR, suggesting that the particle concentration is higher in the AR. Also, considering that the cross section of the AR is 2.2 times that of the FR, it is evident that the particle transport up the FR is much lower than that of the AR, and thus most of the particle transport from the FR to the AR is through the lifter. In this case, the AR riser mass flow was calculated to 4.7 kg/s, while the corresponding FR riser mass flow was only 0.3 kg/s. For the particles used in this test, the terminal velocity is about 0.8 m/s, and the minimum fluidization velocity is about 0.01 m/s. The lifter superficial velocity was about 0.36 m/s; therefore, it is operated as a bubbling fluidized bed in the same manner as a loop-seal. The lifter fluidization can be used to regulate the particle inventory in the FR, and to some extent, the FR riser mass flow. When the lifter fluidization is reduced, the particle inventory in the FR increases and stabilizes at a higher value, which to some degree increases the FR riser mass flow depending on the superficial velocity of the FR riser.

### 3.2 Main Results

The three key performance parameters evaluated are the CO_2_ capture efficiency η*_CC_*, the FR gas conversion efficiency η_gas_, and the FR carbon conversion, *X*_C_. They are summarized in Table 4 and further discussed below.

**Table 4:** Key Performance Indicators for the Different Cases

	key performance indicator		
	CO_2_ capture eff.	FR gas conv. eff.	FR carbon conv.
test case	η_CC_ [%]	η_gas_ [%]	*X*_C_ [%]
wood pellets	95.0	76.4	89.7
milled wood pellets	93.5	79.4	89.3
SRF pellets 1	97.9	79.6	91.3
SRF pellets 2	97.8	80.7	89.9
SRF pellets 3	97.6	81.4	89.8
wood pellets Mn + Ilm.	94.4	85.3	89.9

#### 3.2.1 CO_2_ Capture and FR Gas Conversion Efficiencies

In Figure 9, the CO_2_ capture efficiency and the FR gas conversion efficiency are visualized together with the FR average temperature, the average of the bottom and top FR temperature.

**Figure 9:**
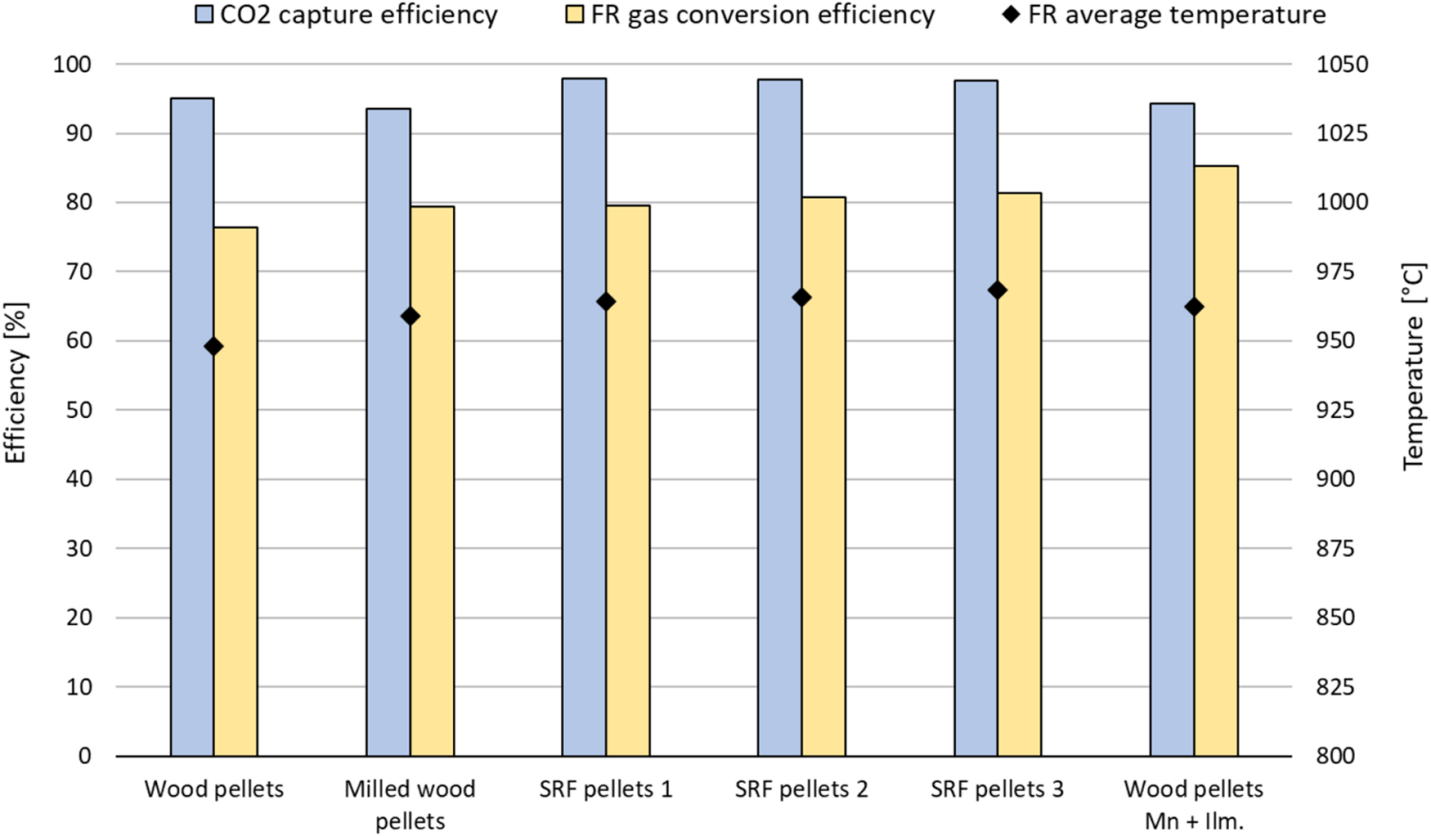
CO_2_ capture efficiency (η_CC_), FR gas conversion efficiency (η_gas_), and FR average temperature for all cases.

The FR gas conversion efficiency for the reference wood cases using only ilmenite as OC (the first two cases in Figure 9) is in the range 76–79%, which is in accordance with the literature and in the higher end of what has previously been achieved with biomass and ilmenite.^17,18^ The three SRF cases all show slightly higher FR gas conversion efficiency than the wood cases, with values of 80–81%.

The CO_2_ capture efficiency for the two wood cases with ilmenite OC is well above 90%, ranging from 93.5 to 95%. The SRF cases all have even higher CO_2_ capture efficiencies, ranging from 97.5 to 98%, showing that very little carbon is transported from the FR to the AR.

In general, the SRF cases with ilmenite have at least as good performance as the wood cases with ilmenite. They show a slight increase in the FR gas conversion efficiencies, while the CO_2_ capture efficiencies show a clearer improvement.

Ilmenite OC has almost no CLOU (chemical looping oxygen uncoupling) effect; i.e., it will not release oxygen directly to the gas phase in the fuel reactor. On the other hand, manganese oxide materials do have some CLOU effect. Their ability to release oxygen directly to the gas phase in the FR can provide an increase in the FR gas conversion efficiency, especially when converting fuels with a high share of volatiles, such as biomass and SRF. The last case in Figure 9 involves an OC mixture of manganese oxide and ilmenite, where the manganese oxide constitutes 70 wt %. This OC mixture was tested using only biomass as the fuel. The biomass is the same “Wood pellets” as used in the first case in the figure. The “Wood pellets Mn + Ilm.” case achieved a FR gas conversion efficiency of 85.3%, compared to 76.4% for the “Wood pellets” case that was done with ilmenite only. This is an 8.9%-point increase which represents a large improvement in the performance. It has about the same CO_2_ capture efficiency, 94.4 vs 95.0%, and the same FR carbon conversion *X*_C_ (cf. Section 2.4.1), 89.9 vs 89.7%. The solid fuel feed rate is also the same. This means that of the carbon fed with solid fuel, the same amount goes to the AR and disappears as CO_2_ in the AR exhaust, and the same amount of fuel is converted to gas in the FR by devolatilization and gasification. However, the Mn-containing case has a larger ability to fully convert these devolatilization and gasification gases to CO_2_ and H_2_O in the fuel reactor than the “wood pellets” case using only ilmenite. The conditions are similar but not completely the same, as the gas flows and air preheat temperature is fine-tuned for each case attempting to get into stable autothermal condition. This is necessary to avoid unstable conditions such as declining temperatures and fuel conversion, or rising temperatures toward unacceptable levels. As the Mn particles are slightly smaller and lighter than the ilmenite particles, the air flow could be reduced, so the excess air ratio is 1.1 compared to 1.3 in the ilmenite case. The air preheat temperature could also be reduced to 385 °C compared to 675 °C for the ilmenite case, likely because a higher share of the fuel is fully converted in the FR. There is a slightly higher FR temperature in the Mn-containing case (14 °C higher) while the AR temperatures are about the same (within 5 °C). The higher FR temperature should help increase the FR gas conversion efficiency to some degree. However, it is probable that the rather large increase in fuel conversion is mainly a result of the ability of the Mn oxide to release oxygen in gas phase, which should be especially favorable for high volatile fuels.

#### 3.2.2 Carbon Balance and Total Fuel Conversion

Figure 10 shows the sum of carbon leaving the FR as gas and the carbon leaving the AR as gas. Both are given in percentage of the carbon fed with the solid fuel. Carbon out of FR as gas is equal to the FR carbon conversion *X*_C_ times 100% (cf. Section 2.4.1) and is the percentage of carbon fed with the fuel that is leaving the FR in gaseous form after devolatilization, gasification, and combustion. Carbon out of AR as gas is equal to the amount of CO_2_ leaving the system with the AR outlet gas and is equal to (1 - η_CC_) times 100% (cf. Section 2.4.3). A small value for carbon out of AR as a gas is equivalent to having a high CO_2_ capture efficiency.

**Figure 10:**
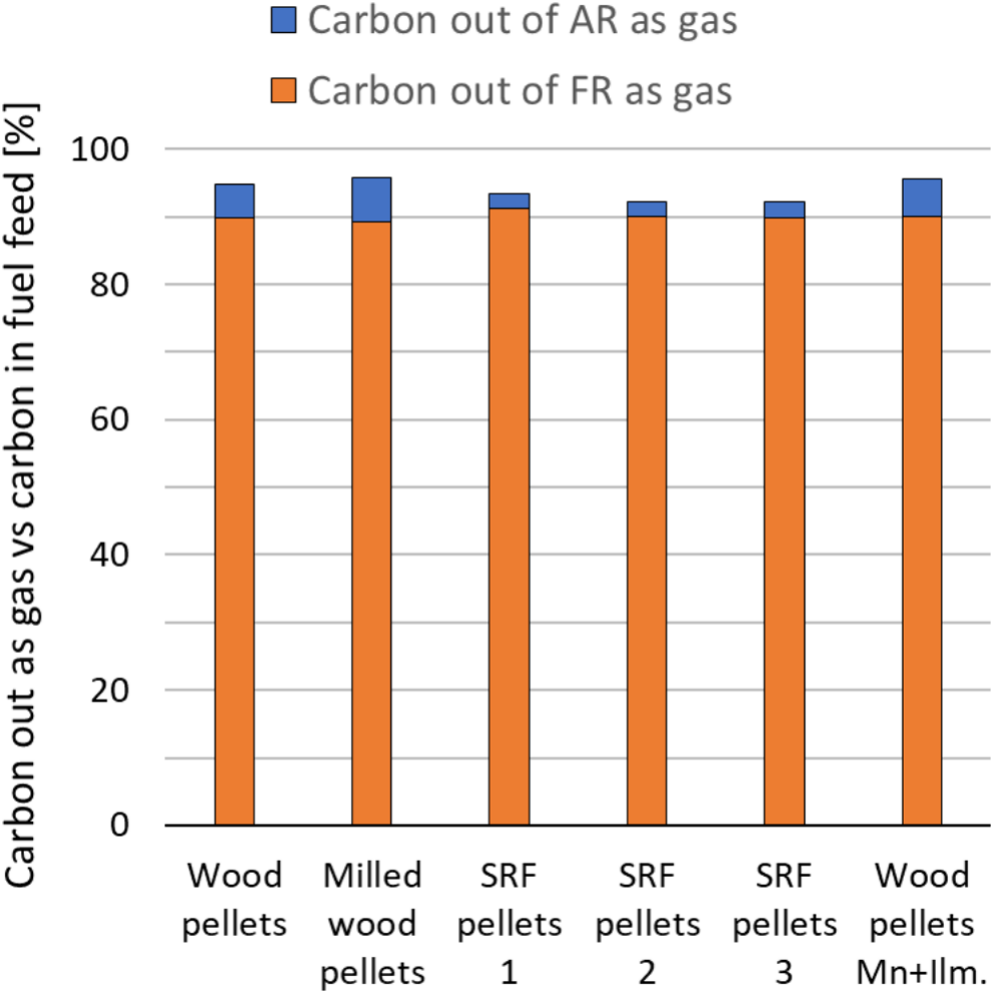
Carbon out of FR as gas plus out of AR as gas as a percentage of carbon fed with the solid fuel.

The carbon balance as calculated here is the difference in percentage between all the carbon fed with solid fuel and the carbon leaving the system as gaseous components (i.e., 100% minus percentage carbon leaving FR + AR as gas). The deviation from 100% implies a loss of particulate carbon leaving the FR, plus any uncertainties in, e.g., the fuel feeding rate. Any such carbon-containing particulates will have to be oxidized in an oxygen-polishing step downstream of the FR, together with unconverted gaseous components. This will increase the real oxygen demand for the oxygen-polishing step compared to the purely gaseous-based oxygen demand calculated according to Section 2.4.2.

Looking at, for example, the wood pellets case, the FR particulate loss seems to be about 5%. This will not imply an increase in oxygen demand of 5%-points, but about 3.7%-points, as the oxygen demand accounts for hydrogen leaving the FR. However, it is difficult to evaluate this accurately without collecting and quantifying all carbon-containing particles leaving the FR.

The SRF cases generally show a lower sum of carbon out as gas from the FR plus AR than the wood cases. This could imply a higher loss of particulates. To assess this, a particle size distribution (PSD) and carbon analysis was performed on particle samples from the FR exhaust for a wood pellets case and an SRF case. The sampling was done as described in Section 2.1. The PSDs (Figures 11 and 12) are as expected and generally equal in both cases, with particle sizes being reduced when moving downstream the exhaust line from the reactor to the scrubber. The loss of smaller particles with the exhaust gas shifts the FR reactor particles toward larger size compared to the fresh OC, as is clearly seen in Figure 12. Figures 13 and 14 show the measured carbon contents of the samples. There is a considerably higher amount of solid carbon in the FR exhaust samples after SRF operation than after wood pellets operation. And especially in the scrubber. The carbon particles that flow all the way to the scrubber must be very small, indicating that they are almost soot-like particles. They could be oxidized in the FR exhaust afterburner, but the design of the afterburner on this unit is not very efficient due to improper mixing and high heat losses.

**Figure 11:**
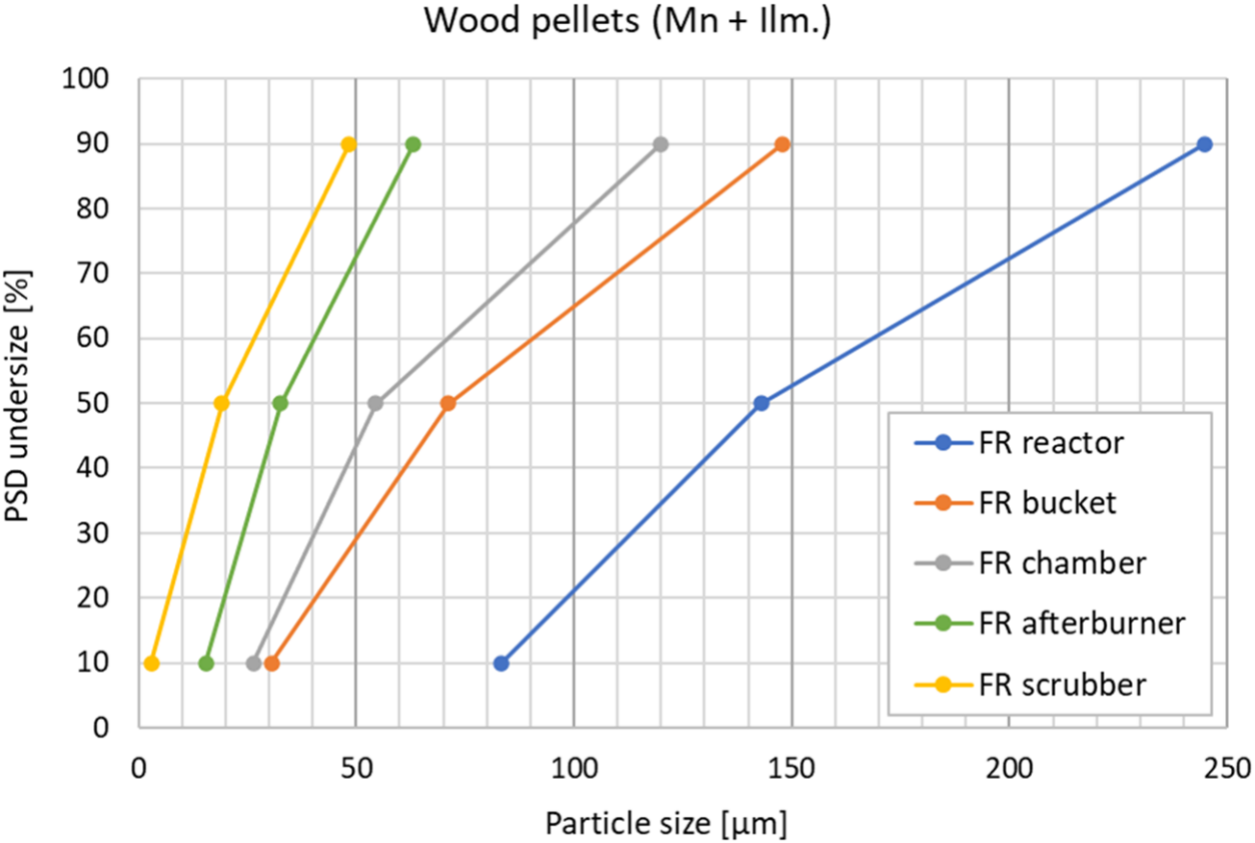
Particle size distribution (PSD) of FR particle samples after wood pellets operation.

**Figure 12:**
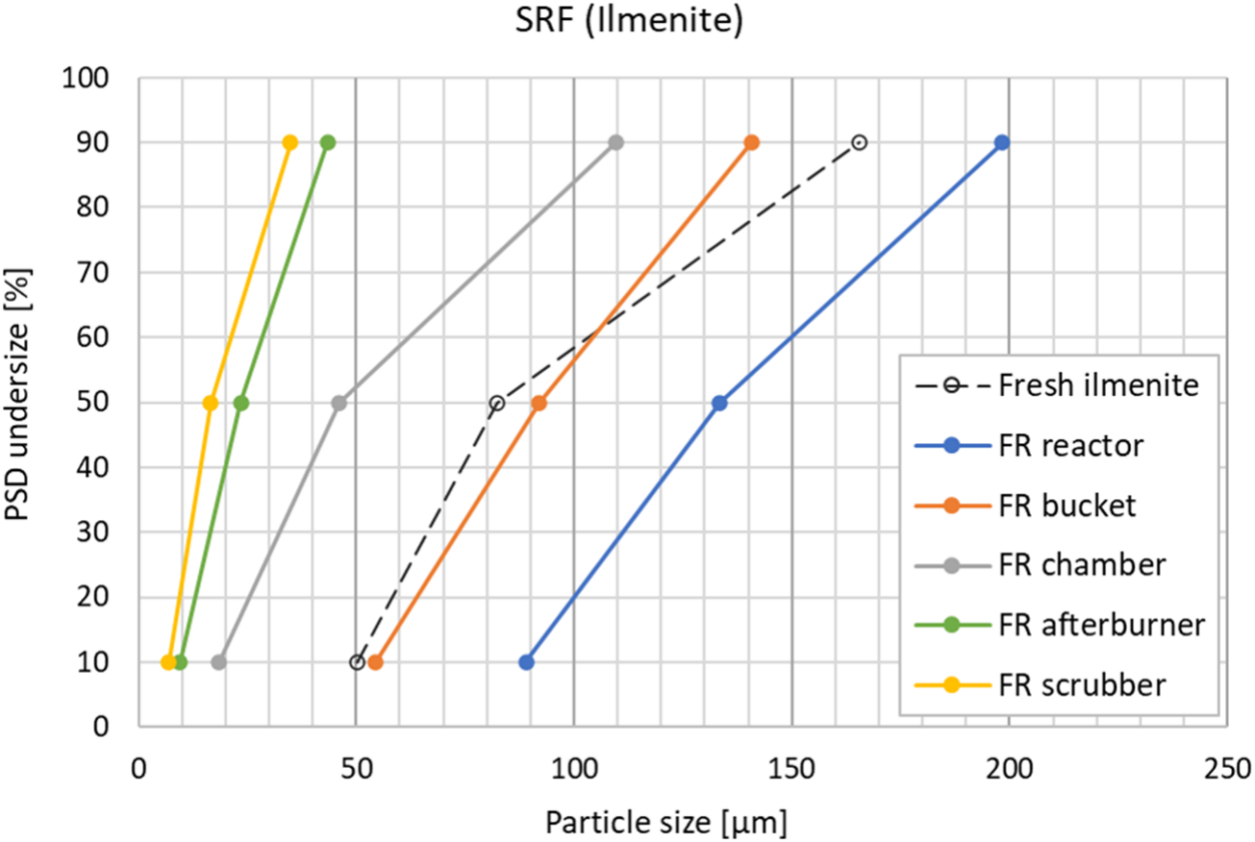
Particle size distribution (PSD) of the FR particle samples after SRF operation.

**Figure 13:**
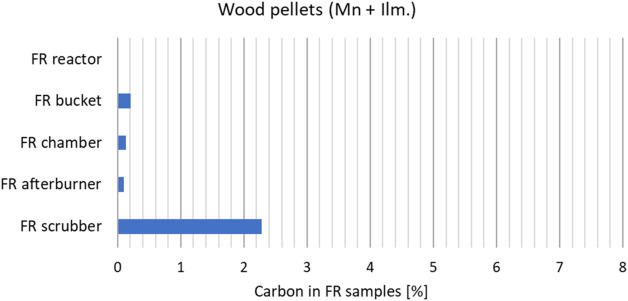
Measured carbon (wt %) in the FR particle samples after wood pellets operation.

**Figure 14:**
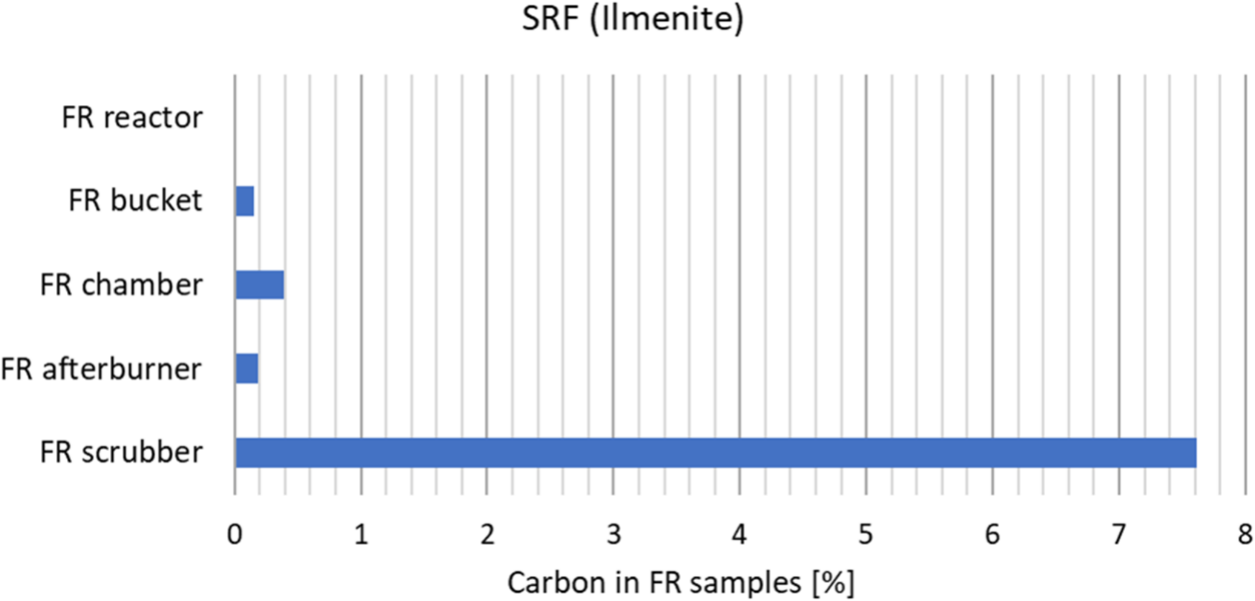
Measured carbon (wt %) in the FR particle samples after SRF operation.

In essence, the use of SRF seems to produce more carbon particulates that leave with the FR exhaust than the wood cases. And the particulates seem to be very small.

When the particle samples were taken out of the reactors, no signs of detrimental agglomeration were detected. Both the ilmenite and the ilmenite/Mn mixture were still free-flowing single particles like when unused. However, it should be noted that the SRF test was performed for just one long day, with 7 h of continuous operation in CLC mode. The possible influence from the fuel on the oxygen carrier will most likely need a longer time in operation. The present work was a first test of the feasibility of using SRF waste-derived fuel in the CLC unit. A longer test period will have to be arranged in the next stage.

#### 3.2.3 NO*_x_* and the Fate of Fuel Nitrogen

Since the AR temperature in a CLC process is too low for significant formation of thermal NOx, and gaseous N_2_ should not enter the FR, the only concern is what happens with the nitrogen entering the FR through the fuel. Nitrogen-containing gas species such as NO, NO_2_, and NH_3_ were measured by FTIR but are unfortunately not available for the relevant time periods due to several challenges with the sampling equipment. However, results from a recent test with the same type of wood pellets and pellets made from treetops and branches with a higher N content, also using ilmenite as OC, can be used as an illustration. The N-content of this fuel (0.58 wt %) is higher than the wood pellets (0.1 wt %) but lower than the SRF (1.3 wt %), so the effects of increased N-content should be even higher for the SRF fuel. Figure 15 shows the NO and NH_3_ concentrations in the FR exhaust when switching from the lower N fuel to the higher-N fuel.

**Figure 15:**
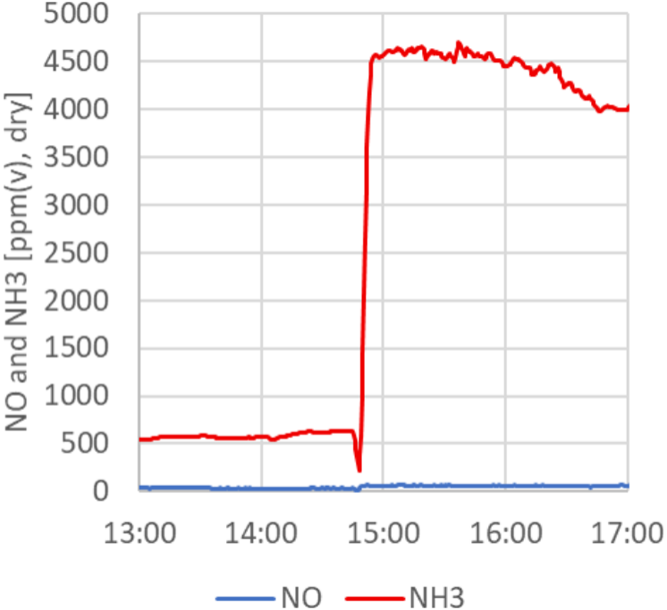
NO and NH_3_ concentrations in FR exhaust when switching from a low nitrogen fuel (Arbacore wood pellets, <0.1 wt % N) to a higher nitrogen fuel (pellets of tops and branches, 0.58 wt % N) at 14:50.

The concentration of NH_3_ is high and constitutes the major part of the nitrogen released from the fuel nitrogen (about 60–75% of the fuel-N). When switching from the Arbacore wood pellets to the higher fuel-N pellets of tops and branches, the NH_3_ increases from around 600 to 4500 ppm, while the NO concentration increases from around 30 to around 60 ppm. The ability of the ilmenite OC to oxidize the NH_3_ to NO is limited, but not totally absent under these conditions, which is in line with what was found by other authors, e.g., ref 19. However, it is probable that more NO is formed when using OC with a higher CLOU effect, as the manganese oxide Micromax particles. This, together with the NO formation in the downstream oxygen-polishing step, will be topics for future studies.

## 4 Conclusions

In this work, pilot tests with solid recovered waste fuel (SRF) are performed in the 150 kW_th_ autothermal CLC pilot unit at SINTEF Energy Research in Norway. Tests with woody biomass performed in the same unit serve as a reference. The fuel feeding rate is 20 kg/h, equivalent to about 115 kW_th_ based on the lower heating value of the fuel. The CO_2_ capture efficiency is high, well above 95%, and is higher for SRF than biomass, even though this pilot unit does not have a carbon stripper. The fuel reactor (FR) gas conversion efficiency is 80–81% for the SRF cases, which is slightly higher than for the biomass reference cases.

The carbon balance indicates that more fine carbon particulates leave the unit with the FR flue gas in the SRF cases than for biomass. Carbon analysis of particle samples from the flue gas is used to verify the observed difference in carbon balance between the two fuels. The oxygen needed to oxidize carbon particulates leaving with the FR exhaust must be added to the amount found from the gas-based oxygen demand parameter.

When using an OC mixture of 70 wt % manganese oxide and 30 wt % ilmenite, the FR gas conversion efficiency is increased nearly 9%-points as compared to using ilmenite OC alone. This is a large improvement in the performance and is due to the CLOU effect of manganese oxide and the greater ability to fully oxidize gases from devolatilization and gasification of the fuel. This test was only performed with wood pellets, but it is believed that the same effect will also be seen when using SRF. The possibility of increasing the FR gas conversion efficiency with oxygen carrier materials with higher CLOU effect should be investigated further to reduce the need for supplementary oxygen for complete gas conversion in an oxygen-polishing step downstream of the fuel reactor.

In general, the SRF cases with ilmenite show at least as good performance as the wood cases with ilmenite. Both fuels are well suited for CLC, stable autothermal operation can be achieved, and the carbon capture efficiency can be high even without a carbon stripper between the fuel and air reactor. A possible omission of a carbon stripper will save both investment and operational costs.

The possible use of cheaper feedstocks, involving waste-derived fuels and other biogenic residuals, can be especially interesting in CLC, where the “dirtier” side of the fuel reactor is separated from the most exotherm and heat-producing side in the air reactor. This can provide higher energetic efficiency than conventional waste-to-energy conversion processes since high-temperature corrosion issues will be less of a problem. At the same time, CLC provides a concentrated CO_2_ stream that is ready for utilization or permanent storage, contributing to carbon dioxide removal.
